# Structural aspects of intermolecular interactions in the solid state of 1,4-dibenzylpiperazines bearing nitrile or amidine groups

**DOI:** 10.1107/S2052520614013754

**Published:** 2014-09-18

**Authors:** Mateusz Rezler, Teresa Żołek, Irena Wolska, Dorota Maciejewska

**Affiliations:** a Department of Organic Chemistry, Faculty of Pharmacy, Medical University of Warsaw, Banacha 1, Warsaw 02 097, Poland; b Department of Crystallography, Faculty of Chemistry, Adam Mickiewicz University, Grunwaldzka 6, Poznań 60 780, Poland

**Keywords:** pharmaceuticals, solid-state study, density functional theory, spanning X-ray diffraction

## Abstract

The crystal structures of the title 1,4-bis(4-cyanobenzyl)piperazine (1) and 1,4-bis(4-amidinobenzyl)piperazine tetrahydrochloride tetrahydrate (2) are reported. Compound (1) crystallizes in the triclinic space group  and compound (2) in the monoclinic space group *P*2_1_ */n*. In both (1) and (2) the asymmetric unit contains one half of the molecule because the central piperazine rings were located across a symmetry center. The packing of both molecules was dominated by hydrogen bonds. The crystal lattice of (1) was formed by weak C—H⋯N and C—H⋯π interactions. The crystal structure of (2) was completely different, with cations as well as chloride anions and water molecules taking part in intermolecular interactions. Single-crystal X-ray diffraction studies combined with density functional theory (DFT) calculations allowed the characterization of the intermolecular interactions in those two systems having different types of very strong electrophilic groups: non-ionic nitrile and ionic amidine. Chemical shift data from ^13^C CP/MAS (Cross Polarization Magic Angle Spinning) NMR spectra were analyzed using the different procedures for the theoretical computation of shielding constants.

## Abstract 2

X-ray diffraction analyses for new pentamidine analogs are presented: 1,4-bis(4-cyanobenzyl)piperazine (1) crystallizes in the triclinic space group (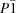) and 1,4-bis(4-amidinobenzyl)piperazine tetrahydrochloride tetrahydrate (2) in the monoclinic space group (*P*2_1_/*n*) revealing a complex system of hydrogen bonds for (2).

## 1. Introduction   

The increasing demands of the pharmaceutical industry for rapid molecular structure determination of pharmaceutical solids have prompted the development of joint analysis methods spanning X-ray diffraction, ^13^C CP/MAS NMR and molecular modeling. The solid-state form of a drug can have a dramatic effect on its bioavailability and physical properties, and the regulatory approval for many drugs is granted only for the defined polymorph (Barrett *et al.*, 2013 ▶; Geppi *et al.*, 2008 ▶; Pereira Silva *et al.*, 2011 ▶). Moreover, because the existence of solvates (called pseudopolymorphs) is an abiding problem in pharmaceutical chemistry (the solid-state form of a solvate can also be treated as the separate form of the drug), it is important to structurally characterize these. Solid-state structural studies of new substances which are designed as potential chemotherapeutic agents has also aroused great interest (Harris, 2007 ▶). Hydrogen bonds are crucial to the interactions between biomolecules, with the macromolecular target and their analysis contributing to expanding the information about biomolecular interactions.

The molecules analyzed in this investigation can be considered as pentamidine analogs and are of interest because of their potential as chemotherapeutic agents against pneumocystis pneumonia (PCP) caused by the fungus *Pneumocystis jiroveci* in patients with compromised immune systems (Ponce *et al.*, 2010 ▶; Furrer *et al.*, 1999 ▶; Maini *et al.*, 2013 ▶) or as anticancer and antimicrobial agents (Barrett *et al.*, 2013 ▶).

The screening of new pentamidine analogs led to the selection of less toxic and highly active molecules (with *in vitro* IC_50_ values as low as 0.002 µM compared with 0.5 µM for pentamidine) which contain functional groups such as the piperazine ring that increase the rigidity of the molecule (Vanden Eynde *et al.*, 2004 ▶; Huang *et al.*, 2009 ▶; Cushion *et al.*, 2004 ▶, 2006 ▶; Mitsuyama *et al.*, 2008 ▶). The intermolecular interactions in the solid state of piperazine-type pentamidine analogs were not examined, and the results obtained here could be useful in the future explanation of their biological features.

**Figure 1:**
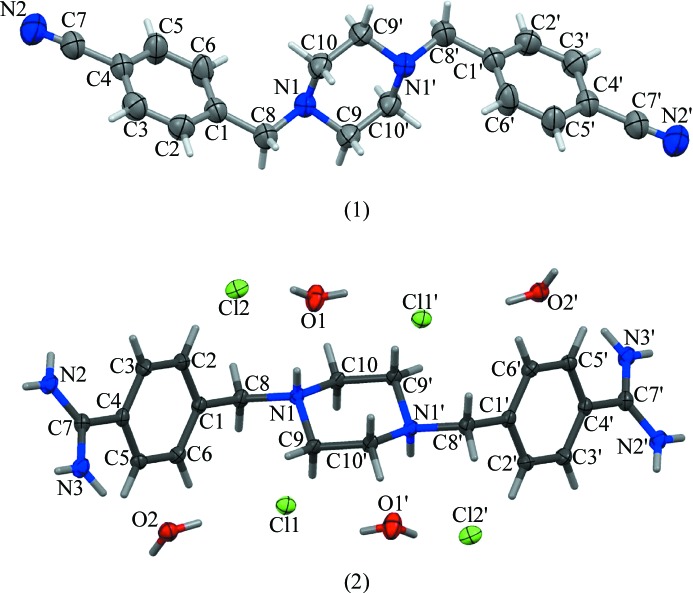
Molecular conformation and atom-numbering for (1) and (2). Displacement ellipsoids are drawn at 50% probability for non-H atoms. The molecules lie across inversion centers according to the symmetry operations 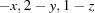 in (1) and 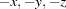 in (2).

In this investigation we analyzed and compared the solid-state structures of new pentamidine analogs containing the piperazine moiety (Fig. 1 ▶), *i.e.* 1,4-bis(4-cyanobenzyl)piperazine (1) and 1,4-bis(4-amidinobenzyl)piperazine tetrahydrochloride tetrahydrate (2) with particular attention paid to hydrogen bonding, using different methods: single-crystal X-ray diffraction analyses combined with molecular modeling and ^13^C CP/MAS NMR spectroscopy. The bis-nitrile compound (1) is an intermediate for the synthesis of bis-amidine (2) *via* the Pinner reaction (Pinner & Klein, 1877 ▶). Diffraction-quality single crystals of bis-amidines are difficult to grow, and the crystal structures of few bis-amidines related to pentamidine have been reported (Maciejewska *et al.*, 2006 ▶; Lowe *et al.*, 1989 ▶; Srikrishnan *et al.*, 2004 ▶; Donkor *et al.*, 1995 ▶). Some authors used the information obtained for the crystals of bis-nitriles to explain the properties of bis-amidines (Cui *et al.*, 2003 ▶). (The authors designed the inhibitors of key enzymes in the coagulation cascade, and to experimentally check the geometry of the amidine inhibitors they determined their cyanosynthetic intermediates by X-ray diffraction.) In this work we show that such an approach to the problem is inappropriate due to the limited similarity between those two groups.

## 2. Experimental   

### 2.1. Chemistry   

All chemicals were purchased from major chemical suppliers as high or the highest purity grade and were used without any further purification. The solvents, K_2_CO_3_, HCl, NaOH and ammonia, were obtained from POCH (Gliwice, Poland). The substrates 4-cyanobenzyl bromide and piperazine were obtained from Alfa AESAR (Karlsruhe, Germany). The scheme below[Chem scheme1] presents the synthetic route to bis-amidine (2) *via* bis-nitrile (1). In the first step 1,4-bis(4-cyanobenzyl)piperazine (1) was prepared by a modification of the procedure given by Gruenenthal (2008 ▶). The Pinner reaction of (1) to form the bis-amidine (2) was conducted for 2 weeks due to the poor solubility of (1) in ethanolic HCl.
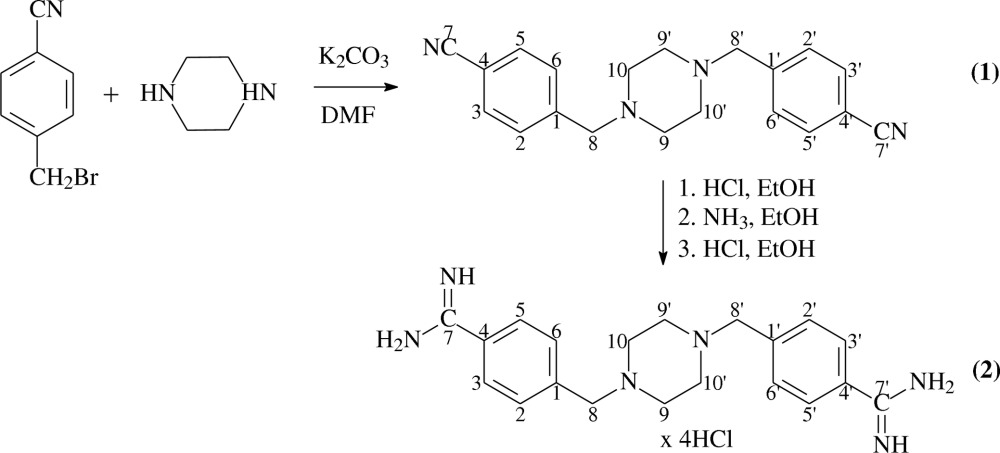


Compound (1) was mentioned in the paper by Spychała (1999 ▶), but the detailed synthetic and spectral information was not given and therefore it is described in §2.1.1. Melting points were determined with an Electrothermal 9001 digital melting point apparatus. Elemental analyses were performed on a Vario EL III CHNS element analyzer, and were averaged from two independent determinations. Chemical shifts (p.p.m.) in CDCl_3_ (1) or DMSO-*d*_6_ (2) were referenced to tetramethylsilane (TMS) .

#### 2.1.1. 1,4-Bis(4-cyanobenzyl)piperazine (1)   

4-Cyanobenzyl bromide (19.6 g 0.1 mol), K_2_CO_3_ (13.8 g, 0.1 mol), piperazine (4.3 g, 0.05 mol) and DMF (235 ml) were stirred for 1 h at room temperature and then stirred and heated for 5 h at 353–363 K. The reaction mixture was cooled to room temperature, ice-water (700 ml) was added, and stirring was continued for 0.5 h at 272–278 K. The white precipitate was filtered off, washed with cold water (2 × 400 ml) and dried *in vacuo*. The crude product was crystallized from acetone to give 27.2 g (86%) of fine colorless crystals of (1). M.p. 479.5–480.5 K; C_20_H_20_N_4_ (*M*_r_ = 316): calc. C 75.95, H 6.33, N 17.72%; found C 75.99, H 6.36, N 17.68%. ^1^H NMR (299.87 MHz, CDCl_3_): δ = 2.48 (br s, 8H, 9-CH_2_, 9′-CH_2_, 10-CH_2_, 10′-CH_2_), 3.56 (s, 4H, 8-CH_2_, 8′-CH_2_), 7.43–7.46 (d, *J* = 8.1 Hz, 4H, 2-CH, 2′-CH, 6-CH, 6′-CH), 7.59–7.62 (pd, *J* = 8.1 Hz, 4H, 3-CH, 3′-CH, 5-CH, 5′-CH) p.p.m. ^13^C NMR (50.28 MHz, CDCl_3_): δ = 53.24 (C9, C9′, C10, C10′), 62.54 (C8, C8′), 111.15 (C4, C4′), 119.12 (C7, C7′), 129.71 (C2, C2′, C6, C6′), 132.32 (C3, C3′, C5, C5′), 144.22 (C1, C1′) p.p.m.

#### 2.1.2. 1,4-Bis(4-amidinobenzyl)piperazine (2)   

A slurry of 1,4-bis(4-cyanobenzyl)piperazine (1) (1.26 g; 4 mmol) in anhydrous ethanol (40 ml) was saturated with anhydrous HCl at 273–278 K. The contents were stirred in a sealed vessel for 2 weeks at room temperature. The reaction was carried out until the starting material was completely consumed (TLC, IR). The solvent was then removed almost to dryness *in vacuo* at 312 K. The residue was ground with dry diethyl ether (100 ml) until colorless crystals of an unstable intermediate (ethyl imidate) formed; these were quickly filtered off and dried under reduced pressure over NaOH granules.

Dry ethanol (40 ml) was saturated with anhydrous ammonia gas at 273–278 K, the entire amount of ethyl imidate added and the mixture stirred in a sealed vessel for 24 h at room temperature. Ethanol was removed almost to dryness under reduced pressure and a solution of NaOH (1.0 g) in water (40 ml) was added to the residue and stirred for 15 min. The free base, which formed as a fine white precipitate, was filtered, washed thoroughly with water and dried under reduced pressure over anhydrous NaOH granules. The dry powder was washed with chloroform to remove unreacted bis-nitrile, dried again *in vacuo* and mixed with anhydrous ethanol (10 ml), acidified with an excess of ethanolic HCl and refluxed for 0.5 h. After cooling, dry diethyl ether (30 ml) was added slowly with stirring. After a few minutes the colorless crystals were filtered off, washed with dry diethyl ether and dried. Recrystallization from aqueous ethanol gave 1.90 g (84%) of pure bis-amidine (2). M.p. 564–566 K; C_20_H_26_N_6_·4HCl·4H_2_O (*M*_r_ = 568): calc. C 42.25, H 6.69, N 14.79, Cl 25.00%; found C 42.31, H 6.77, N 14.42, Cl 24.57%. ^1^H NMR (299.87 MHz, DMSO-d_6_): δ = 3.48 (br s, 10H, 9-CH_2_, 9′-CH_2_, 10-CH_2_, 10′-CH_2_, 2NH), 4.49 (s, 4H, 8-CH_2_, 8′-CH_2_), 7.94 (bs, 8H, 2-CH, 2′-CH, 6-CH, 6′-CH, 3-CH, 3′-CH, 5-CH, 5′-CH), 9.38 (s, 4H, 2NH_2_), 9.56 (s, 4H, 2NH_2_) p.p.m. ^13^C NMR (50.28 MHz, DMSO-d_6_): δ = 48.03 (C9, C9′, C10, C10′), 57.84 (C8, C8′), 128.36 (C3, C3′, C5, C5′), 128.51 (C4, C4′), 131.61 (C2, C2′, C6, C6′), 131.24 (C1, C1′), 164.98 (C7, C7′) p.p.m.

### 2.2. Crystallography   

Crystals of (1) suitable for X-ray analysis were grown by slow evaporation from acetone, and crystals of (2) were grown by slow evaporation from 96% ethanol and a few drops of water (which were added to the ethanol mixture until its homogeneity was achieved). Diffraction data were collected on an Oxford Diffraction KM4 CCD diffractometer using Mo *K*α radiation at room temperature for (1) and on an Oxford Diffraction SuperNova CCD diffractometer using Cu *K*α radiation at 130 K for (2). Data reduction was carried out using *CrysAlis Pro* (Agilent, 2011 ▶, 2012 ▶) for (1) and (2), respectively.

The unit-cell parameters were determined by least-squares treatment of the angles of the highest-intensity reflections chosen from all the experiments. The structures were solved by direct methods using *SHELXS*97 and refined on *F* ^2^ by full-matrix least-squares with *SHELXL*97 (Sheldrick, 2008 ▶). The function 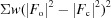 was minimized with 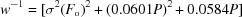 for (1) and 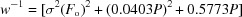 for (2), where 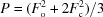. For (1) an empirical extinction correction was also applied according to the formula 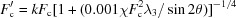, and the extinction coefficient χ was equal to 0.08 (1).

All non-H atoms were refined with anisotropic displacement parameters. The coordinates of the H atoms of (1) and the majority of hydrogen positions of (2) were generated geometrically. In (2) the hydrogen at N1 and the H atoms of water molecules were found on the difference map. Then the H atom involved in hydrogen bonds in (1) and all the H atoms in (2) were refined isotropically. The other atoms were refined as a riding model with *U*_iso_(H) = 1.2*U*_eq_(carrier atom). All the details of the measurement, crystal data and structure refinement are given in Table 1 ▶. 1

### 2.3. Molecular modeling   

**Figure 2:**
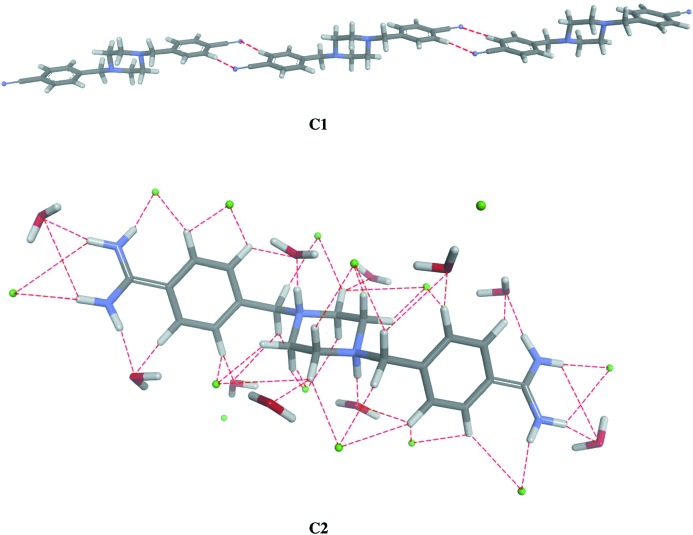
Views of the clusters *C*1 and *C*2 with hydrogen-bonding scheme analyzed for molecules (1) and (2).

Structural optimizations were performed at the density functional theory (DFT) level with B3LYP/6-311(d,p) hybrid functional, and the locations of the true minima were confirmed by vibrational analysis using *GAUSSIAN*09 (Frisch *et al.*, 2009 ▶). The crystal atomic coordinates were used as the starting point for DFT computations. To investigate the intermolecular interactions in the solid state (Gholivand *et al.*, 2013 ▶), the optimization of H atoms positions was performed for the cluster *C*1 built up from three molecules of (1). The target molecule of (1) was surrounded by two neighboring molecules from one layer which were connected to each other *via* two C3—H3*A*⋯N2 hydrogen bonds with a donor–acceptor distance of 3.629 (2) Å (Fig. 2 ▶). For compound (2) cluster *C*2 was built up from one cation of molecule (2) surrounded by 14 chloride anions and ten water molecules (Fig. 2 ▶), because compound (2) did not form hydrogen bonds directly with another molecule of (2). All hydrogen-bond parameters are given in Table 2 ▶. The positions of the H atoms were optimized, while the other atoms were kept fixed during the optimizations. This approach allowed us to perform the analyses of the central molecule when the neighboring molecules were present. The hydrogen-bonding energies were calculated for compound (1) using the equation: *E*_Hbond_ = ½(*E*_cluster_ − *E*_two_ − *E*_one_), taking into account the two hydrogen bonds C3—H3*A*⋯N2 formed in the layer. *E*_two_ is composed of two neighboring molecules at the left-hand side of Fig. 2 ▶, and *E*_one_ is the remaining part of *C*1. For compound (2) the average energy of hydrogen bonds was calculated based on the energy difference between the hydrogen-bonded cluster and its fragments as represented by the equation: *E*_Hbond_ = 1/28(*E*_cluster_ − *E*_anions+water_ − *E*_one_).

The isotropic ^13^C shielding constants σ (p.p.m.) for (1) and (2) in the solid state were computed with the GIAO (gauge including the atomic orbital) method using *GAUSSIAN*09 (Frisch *et al.*, 2009 ▶) at the DFT level with B3LYP/6-311(d, p) hybrid functional. For the assignment of ^13^C CP/MAS NMR resonances, the structures obtained by X-ray diffraction were optimized prior to chemical shielding calculations using four different procedures:

(i) the positions of all atoms in (1) and (2) were fully optimized;
(ii) the positions of the non-H atoms were fixed, but the H atoms were allowed to move in (1) and (2);
(iii) the positions of all atoms in the clusters *C*1 and *C*2 were optimized, and the shielding constants were analyzed for the target molecules (1) and (2);
(iv) the positions of the non-H atoms were fixed in clusters *C*1 and *C*2, and the shielding constants were analyzed for the target molecules (1) and (2).

### 2.4. NMR spectra measurements   

Solution ^1^H NMR and ^13^C NMR spectra were recorded at 298 K on a Varian NMRS-300 spectrometer and standard Varian software was employed. Solid-state ^13^C CP/MAS NMR spectra were recorded on a Bruker Avance DMX 400 spectrometer at 100.62 MHz using a 4 mm diameter zirconia rotor. Conventional single-contact ^1^H → ^13^C cross-polarization (CP) with reversal of spin temperature in the rotating frame, and high proton decoupling during signal acquisition were performed. The acquisition conditions for ^13^C CP/MAS NMR were: pulse duration 2.5 µs; contact time 4 ms; repetition time 48 s for (1) and 50 s for (2); spectral width 24 kHz; spinning speed 8 kHz. Chemical shifts δ (p.p.m.) were referenced to TMS.

## 3. Results and discussion   

### 3.1. X-ray structure analysis   

The crystal and molecular structures of 1,4-bis(4-cyanobenzyl)piperazine (1) and 1,4-bis(4-amidinobenzyl)piperazine tetrahydrochloride (2) were determined by single-crystal X-ray diffraction. A perspective view (Macrae *et al.*, 2006 ▶) of the molecular conformations of (1) and (2), together with the atom-numbering scheme, is illustrated in Fig. 1 ▶. Hydrogen-bonding parameters are listed in Table 2 ▶, and selected bond lengths, bond angles and torsion angles are listed in Table 3 ▶.

The results show that the compounds 1,4-bis(4-cyanobenzyl)piperazine (1) and 1,4-bis(4-amidinobenzyl)piperazine tetrahydrochloride tetrahydrate (2), the piperazine-derived analogs of pentamidine, crystallize in the triclinic space group 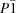 and monoclinic space group *P*2_1_/*n*, respectively. In both (1) and (2), the asymmetric units contain one half of the molecule because the central piperazine rings are located across symmetry centers according to the symmetry operators (−*x*, 2 − *y*, 1 − *z*) in (1) and (−*x*, −*y*, −*z*) in (2). Additionally, in the asymmetric unit of (2) there are two chloride ions and two molecules of water. As a consequence, we found the tetrachloride salt of this derivative in the lattice. The structure of (1) consists of two cyanobenzyl groups which are joined by the piperazine ring which adopts the expected chair conformation. The cyanobenzyl moiety is almost planar with a maximum deviation of 0.045 (1) Å for C8. The orientation of the piperazine ring with respect to the cyanobenzyl fragment is characterized by C1—C8—N1—C9 and C6—C1—C8—N1 torsion angles of −168.6 (1) and 26.0 (2)°, respectively.

In (2) the benzyl group is essentially planar with a maximum deviation of 0.011 (1) Å for C2. The C7 atom from the amidine group was found to be coplanar with the above fragment and the N2 and N3 atoms of this group were displaced by 0.556 (2) and −0.653 (2) Å, respectively. The location of the amidine group with respect to the benzene ring can also be characterized by the C3—C4—C7—N2 and C3—C4—C7—N3 torsion angles of −31.6 (2) and 147.1 (1)°, respectively. The central piperazine ring adopts a chair conformation and its orientation with respect to the benzyl fragment moiety is characterized by C1—C8—N1—C9 and C6—C1—C8—N1 torsion angles of 66.5 (1) and 100.0 (1)°, respectively.

**Figure 3:**
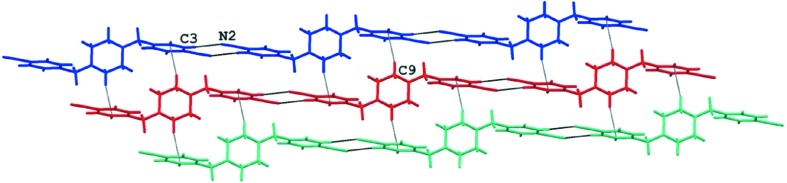
The chains of the molecules of (1) within a layer.

The packing of both molecules is dominated by hydrogen bonds (Table 2 ▶). In the crystal of (1), the nitrile groups take part in intermolecular C3—H3*A*⋯N2 (−*x* + 2, −*y* + 1, −*z* + 2) hydrogen bonds which link the molecules into extended chains. The molecules are further organized into layers parallel to the (011) plane *via* weak C9—H9*A*⋯π(*x* − 1, *y*, *z*) contacts (Figs. 3 ▶ and 4 ▶). We also found such a participation of the nitrile substituents in the hydrogen bonds for other analogs of pentamidine (Żabiński *et al.*, 2007 ▶, 2010 ▶; Maciejewska *et al.*, 2008 ▶).

**Figure 5:**
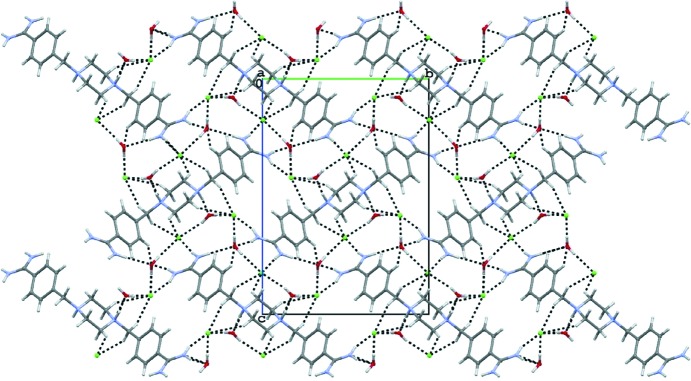
Projection of the crystal structure of (2) along the *a* axis.

The crystal structure of (2) differs in that the packing involves cations, chloride anions and water molecules. Each cation is surrounded by chloride anions and molecules of water and they are linked by N—H⋯O, N—H⋯Cl, C—H⋯O and C—H⋯Cl hydrogen bonds (see Table 2 ▶ and Fig. 5 ▶). The O atoms of the water molecules also participate in O—H⋯Cl interactions and as a consequence a three-dimensional lattice is obtained. Each water molecule and each chloride anion takes part in intermolecular hydrogen bonds. It is interesting to note that there are no direct hydrogen bonds between neighboring molecules of (2). In contrast, the pentamidine analog with three O atoms in the linker forms direct hydrogen bonds using these atoms as proton acceptors (Maciejewska *et al.*, 2006 ▶; Lowe *et al.*, 1989 ▶; Donkor *et al.*, 1995 ▶) even in the presence of water molecules. The protonated N atoms of the piperazine ring in (2) cannot be involved in intermolecular interactions as proton acceptors, and water molecules serve as both proton donors and proton acceptors, providing the main intermolecular links. So far, X-ray studies of the structurally related bis-amidines did not present detailed analysis of the intermolecular hydrogen bonding (Lowe *et al.*, 1989 ▶) or showed only a few intermolecular hydrogen bonds involving the anions or water molecules (Donkor *et al.*, 1995 ▶; Maciejewska *et al.*, 2006 ▶; Lowe *et al.*, 1989 ▶; Srikrishnan *et al.*, 2004 ▶).

### 3.2. Parameters of hydrogen bonding in the clusters   

The theoretical hydrogen-bond parameters for the target molecules (1) and (2) in clusters *C*1 and *C*2 are presented in Table 4 ▶. The donor–acceptor distances for hydrogen bonds in model clusters are equal to the experimental values since the optimizations were performed only for the H-atom positions. The N—H, C—H and O—H bonds are longer by 0.11–0.16 Å than those obtained from the crystal structure determinations, and as a result the hydrogen–acceptor distances H⋯*A* are shorter, suggesting stronger intermolecular interactions. Theoretical N—H⋯O, O—H⋯Cl and N—H⋯Cl angles are more linear than the crystallographic values.

The computed hydrogen-bonding energy in the *C*1 model cluster between the molecules (1) was equal to −8.8 kJ mol^−1^, which is characteristic of very weak interactions. In the first approximation (the hydrogen bonds presented in Table 4 ▶), the average hydrogen-bonding energy in the *C*2 model cluster between the molecule of (2) and the surrounding water and chloride anions was calculated as −406.8 kJ mol^−1^, a very high value. In the second approximation, we considered the 48 interactions with *d*(H⋯*A*) distances below 3.6 Å (thereby incorporating solvation of the cations by water molecules), and the average intermolecular interaction energy of compound (2) with all surrounding chloride anions and water molecules was −237.3 kJ mol^−1^. The intermolecular bonds connecting bis-nitriles are much weaker than the intermolecular bonds formed by bis-amidines. The short contacts in the bis-nitrile crystal (1) are due to weak, nonpolar interactions, whereas in the bis-amidine crystal (2) they are dominated by hydrogen bonds between water molecules and chloride anions.

### 3.3. Solid-state ^13^C CP/MAS NMR spectra analysis of (1) and (2)   

**Figure 6:**
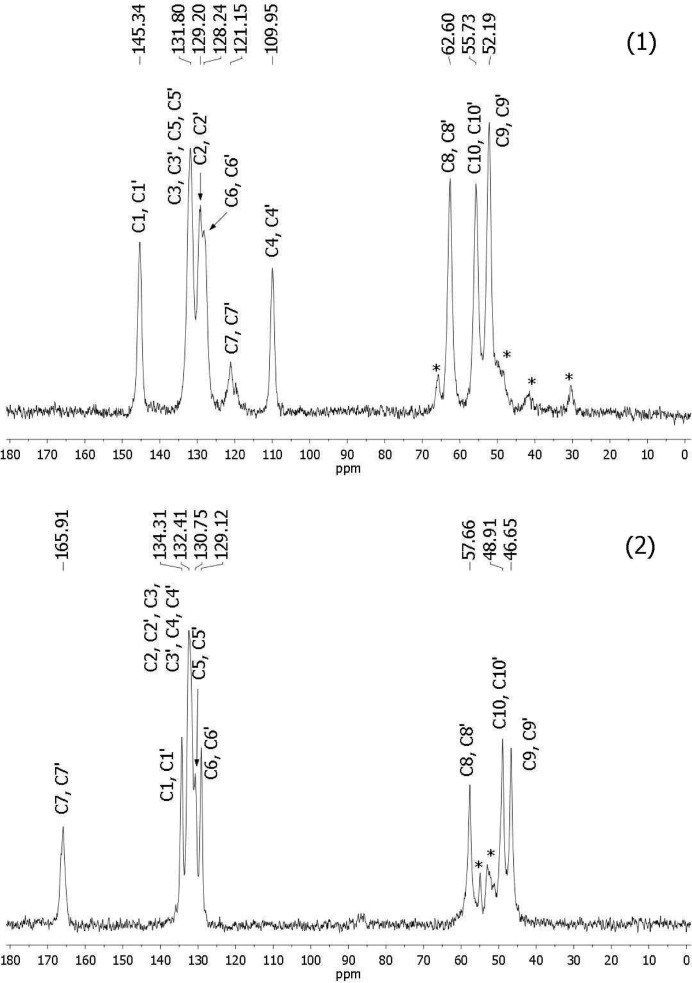
^13^C CP/MAS NMR spectra of compounds (1) and (2). Sidebands are marked with an asterisk.

Solid-state ^13^C CP/MAS NMR spectra of 1,4-bis(4-cyanobenzyl)piperazine (1) and 1,4-bis(4-amidinobenzyl)piperazine (2) are presented in Fig. 6 ▶. The crystals for the ^13^C CP/MAS NMR experiments were collected in the same manner as for the single-crystal X-ray diffraction. In neither spectrum were multiplets observed, *i.e.* we observed a single resonance for each pair of chemically equivalent C atoms. Only for the piperazine ring was an additional signal detected, in accordance with the crystallographic results. Preliminary assignments were carried out on the basis of solution chemical shifts and on the basis of the computed shielding constants obtained for the fully optimized structures of (1) and (2), as described in §2.3 point (i)[Other l1li1]. For molecule (1) the match between the experimental chemical shifts δ and the theoretical shielding constants σ was very close [the correlation coefficient for the linear correlations of σ = *f*(δ) was *R* ^2^ = 0.997 – see the supporting information for more details]. High correlation coefficients were also obtained for the shielding constants calculated by procedures (ii)[Other l1li2] and (iii)[Other l1li3]. The computations correctly predicted higher shielding constants for C9/C9′ than for C10/C10′ in the piperazine ring. As can be seen from Table 5 ▶, significant differences between the isotropic chemical shifts measured in solution and in the solid state were found for C7/C7′ and C3/C3′ which are engaged in hydrogen bonding and for the piperazine C atoms. Surprisingly, the calculations on cluster *C*1 performed using procedure (iv)[Other l1li4] produced the worst correlation, although the correlation coefficient was still high (*R* ^2^ = 0.991). Apparently, the strength of specific solid-state effects is weak, and a simple comparison between the solution and solid-state resonances, and the simple calculation for the isolated molecule is sufficient for structural analysis. The resonances of C3/C3′ and C5/C5′ overlap at 131.8 p.p.m., although the calculation showed higher shielding for C5/C5′. This can be caused by the impact of intramolecular motions of the benzene rings in the solid state which were not considered in the calculations. The resonances of the *ortho* pairs C2/C2′ and C6/C6′ to the piperazine linker were clearly separated. This is a well known phenomenon dependent on intermolecular interactions and the nature of the substituent present at the neighboring C atom. The separation of signals indicated that methylene groups are slightly twisted relative to the benzene ring plane. The resonances of C7/C7′ proximal to N2/N2′ are broadened and split into unequal doublets due to a residual coupling to the quadrupolar ^14^N atom (Olivieri *et al.*, 1987 ▶). For other C atoms proximal to N atoms only some broadening was observed. The ^13^C CP/MAS spectrum of molecule (2) had the same characteristics as the spectrum of (1) in that no multiplets were observed and the C7/C7′ atoms were broadened by a dipolar coupling to the quadrupolar ^14^N nucleus. After preliminary assignment based on the computed shielding constants obtained for the fully optimized structure as described in §2.3 point (i)[Other l1li1], the shielding constants obtained using three different procedures (ii)[Other l1li2], (iii)[Other l1li3] and (iv)[Other l1li4] were compared to the experimental chemical shifts. The correlation coefficients *R* ^2^ for the linear correlations σ = *f*(δ) were within the range 0.973–0.996. The highest correlation coefficient was obtained for procedure (ii)[Other l1li2] – see the supporting information for more details. Procedure (iv)[Other l1li4], which considered cluster *C*2, produced the worst results as well as for cluster *C*1. This observation agreed with our earlier findings for bis-nitriles that the shielding constant computation based on the single molecule structure can be informative for the analysis of solid-state structure based on NMR spectra (Maciejewska *et al.*, 2008 ▶). In the spectrum of (2) separated resonances for all aromatic C atoms were observed. Both C atoms *ortho* and both C atoms *meta* to the amidine group are shielded in different ways. The lack of coplanarity of amidine groups with the benzene ring clearly affects the shielding of these aromatic C atoms. Next we compared the ^13^C resonances in the solid state, δ_solid_, and in solution, δ_solution_ (Table 5 ▶). As can be seen, the highest negative differences between the their chemical shifts were found for C2/C2′, C3/C3′, C7/C7′ and C10/C10′. This was attributed to the engagement of these atoms in the intermolecular interactions with water molecules and chloride anions, which are much stronger than in DMSO solution. Linear pentamidine analogs are molecules of pharmaceutical interest which are rather poorly represented in the crystallographic database: chemical shifts from NMR solid-state spectra of the analyzed compounds have provided valuable information on the solid-state structure to complement the crystallographic data, allowing their hydrogen-bonding patterns to be determined.

## 4. Conclusions   

The structures of 1,4-bis(4-cyanobenzyl)piperazine (1) and 1,4-bis(4-amidinobenzyl)piperazine tetrahydrochloride (2) at 293 and 130 K were solved using single-crystal X-ray diffraction. Compound (1) crystallizes in the triclinic 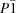 space group, and compound (2) in the monoclinic space group *P*2_1_/*n* with four chloride anions and four H_2_O molecules. The crystal lattice of (1) is formed by weak C—H⋯N and C—H⋯π interactions. The intermolecular interaction energy (evaluated using the equation: *E*_Hbond_ = ½(*E*_cluster_ − *E*_two_ − E_one_) for the former was −8.8 kJ mol^−1^. The crystal lattice formed by (2) is dominated by water molecules and chloride anions, and the average intermolecular interaction energy of compound (2), taking account of all the surrounding chloride anions and water molecules was −237.3 kJ mol^−1^. It is interesting to note that no direct hydrogen bonds exist between neighboring molecules of (2). Our result clearly indicated that structural information and intermolecular interactions in bis-nitriles are not transferable to the structural analysis of bis-amidines. The computation of shielding constants for isolated molecules together with the solid-state spectrum are of considerable value in understanding the solid-state structures of pentamidine analogs.

## Supplementary Material

Crystal structure: contains datablock(s) global, mr-1a, pnt2. DOI: 10.1107/S2052520614013754/bm5065sup1.cif

Structure factors: contains datablock(s) mr-1a. DOI: 10.1107/S2052520614013754/bm5065mr-1asup2.hkl

Structure factors: contains datablock(s) pnt. DOI: 10.1107/S2052520614013754/bm5065pnt2sup3.hkl

Extra tables and figures. DOI: 10.1107/S2052520614013754/bm5065sup4.pdf

CCDC references: 1008046, 1008047

## Figures and Tables

**Figure 4:**
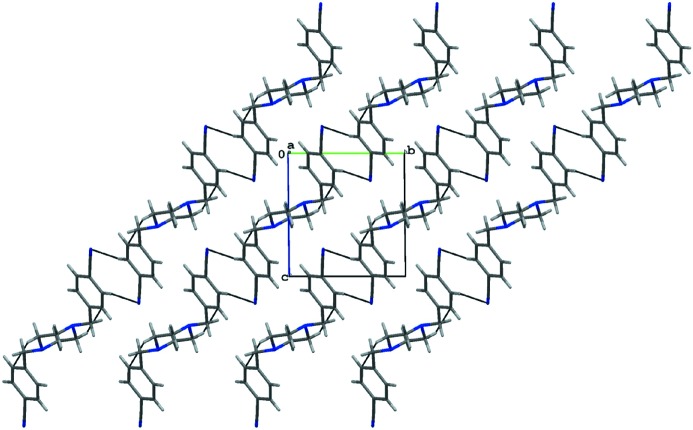
The packing arrangement of (1) showing layers parallel to the (011) plane.

**Table 1:** Crystal data, data collection and structure refinement for (1) and (2)

	(1)	(2)
Crystal data		
Chemical formula	C_20_H_20_N_4_	C_20_H_30_N_6_·4Cl·4H_2_O
*M*_r_	316.40	568.36
Crystal system, space group	Triclinic, *P* 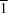	Monoclinic, *P*2_1_/*n*
*T* (K)	293	130
*a*, *b*, *c* (Å)	6.6267 (4), 8.3540 (5), 8.6889 (5)	6.1121 (2), 12.8231 (3), 18.3831 (4)
α, β, γ (°)	83.876 (5), 72.881 (5), 70.676 (5)	90, 99.274 (2), 90
*V* (Å^3^)	433.78 (4)	1421.96 (7)
*Z*	1	2
Radiation type	Mo *K*α	Cu *K*α
μ (mm^−1^)	0.07	4.08
*F*(000)	168	600
Crystal size (mm)	0.5 × 0.4 × 0.35	0.5 × 0.1 × 0.08

Data collection		
Diffractometer	Xcalibur, Sapphire2, large Be window diffractometer	SuperNova, Single source at offset), Atlas diffractometer
Absorption correction	Multi-scan *CrysAlis PRO*	Multi-scan *CrysAlis PRO*
*T*_min_, *T*_max_	0.965, 1.000	0.632, 1.000
No. of measured, independent and observed [*I* > 2σ(*I*)] reflections	6352, 1625, 1409	6224, 2866, 2771
*R*_int_	0.010	0.015
(sin θ/λ)_max_ (Å^−1^)	0.610	0.628

Refinement		
*R*[*F* ^2^ > 2σ(*F* ^2^)], *wR*(*F* ^2^), *S*	0.039, 0.118, 1.05	0.027, 0.073, 1.04
No. of reflections	1625	2866
No. of parameters	114	230
H-atom treatment	H atoms treated by a mixture of independent and constrained refinement	All H-atom parameters refined
Δρ_max_, Δρ_min_ (e Å^−3^)	0.13, −0.12	0.25, −0.21

Computer programs: *CrysAlis PRO* (Agilent, 2011 ▶), *CrysAlis PRO* (Agilent, 2012 ▶), *SHELXS97*, *SHELXL97* (Sheldrick, 2008 ▶), *Mercury* (Macrae *et al.*, 2006 ▶).

**Table 2:** Hydrogen-bonding geometry (Å, °) for (1) and (2)

*D*—H⋯*A*	*d*(*D*—H)	*d*(H⋯*A*)	*d*(*D*⋯*A*)	∠(*D*H*A*)
(1)				
C3—H3*A*⋯N2^i^	0.99 (2)	2.65 (2)	3.627 (2)	166 (1)
				
(2)				
N1—H1⋯O1^ii^	0.90 (2)	1.79 (2)	2.687 (2)	173 (2)
N2—H2*B*⋯O2^vi^	0.85 (2)	2.05 (2)	2.887 (1)	170 (2)
N2—H2*C*⋯Cl2^iv^	0.86 (2)	2.35 (2)	3.189 (1)	166 (2)
N3—H3*C*⋯O2^ii^	0.88 (2)	2.07 (2)	2.941 (1)	175 (2)
N3—H3*B*⋯Cl1^vi^	0.86 (2)	2.43 (2)	3.285 (1)	172 (1)
O1—H1*AW*⋯Cl1^iii^	0.81 (2)	2.27 (2)	3.067 (1)	167 (2)
O1—H1*BW*⋯Cl2^i^	0.84 (3)	2.27 (3)	3.098 (1)	171 (2)
O2—H2*BW*⋯Cl1	0.86 (2)	2.25 (2)	3.088 (1)	163 (2)
O2—H2*AW*⋯Cl2^v^	0.84 (2)	2.26 (3)	3.094 (1)	173 (2)
C8—H8*A*⋯Cl1	0.98 (2)	2.77 (2)	3.678 (1)	155 (1)
C8—H8*B*⋯Cl2^i^	0.95 (2)	2.64 (2)	3.546 (1)	161 (1)
C6—H6*A*⋯O2	0.94 (2)	2.64 (2)	3.422 (2)	141 (1)
C9—H9*A*⋯Cl1^ii^	0.94 (2)	2.70 (2)	3.471 (1)	140 (1)
C10—H10*B*⋯Cl2^i^	0.96 (2)	2.85 (2)	3.723 (1)	151 (1)

Symmetry codes: for (1): (i) 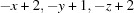; for (2): (i) 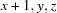; (ii) 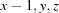; (iii) 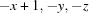; (iv) 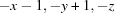; (v) 
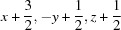
; (vi) 
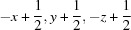
.

**Table 3:** Selected bond lengths (Å), valence angles (°) and torsion angles (°) for (1) and (2)

	(1)	(2)
C4—C7	1.440 (2)	1.483 (2)
C1—C8	1.509 (2)	1.506 (2)
N1—C8	1.458 (2)	1.515 (2)
C2—C1—C8	119.3 (1)	119.9 (1)
C1—C8—N1	114.3 (1)	110.9 (1)
C8—N1—C10	111.1 (1)	110.5 (1)
C6—C1—C8—N1	26.0 (2)	100.0 (1)
C1—C8—N1—C10	71.1 (1)	171.2 (1)
C5—C4—C7—N3	–	−32.4 (2)
C3—C4—C7—N3	–	147.2 (1)

**Table:** 

*D*—H⋯*A*	*d*(*D*—H)_*C*1_	*d*(*D*—H)_gas_	*d*(H⋯*A*)_*C*1_	*d*(*D*⋯*A*)_*C*1_	∠(*D*H*A*)_*C*1_
(1)					
C3—H3*A*⋯N2	1.083	1.083	2.56	3.628	171

**Table:** 

*D*—H⋯*A*	*d*(*D*—H)_*C*2_	*d*(*D*—H)_gas_	*d*(H⋯*A*)_*C*2_	*d*(*D*⋯*A*)_*C*2_	∠(*D*H*A*)_*C*2_
(2)					
N1—H1⋯O1	1.070	1.026	1.62	2.688	177
N2—H2*B*⋯O2	1.016	1.015	1.91	2.886	168
N2—H2*C*⋯Cl2	1.029	1.010	2.17	3.189	172
N3—H3*C*⋯O2	1.035	1.011	1.92	2.942	174
N3—H3*B*⋯Cl1	1.011	1.015	2.27	3.285	178
O1—H1*AW*⋯Cl1	0.969	–	2.10	3.067	177
O1—H1*BW*⋯Cl2	0.974	–	2.12	3.097	179
O2—H2*BW*⋯Cl1	0.976	–	2.12	3.088	176
O2—H2*AW*⋯Cl2	0.967	–	2.13	3.093	175
C8—H8*A*⋯Cl1	1.093	1.091	2.66	3.680	162
C8—H8*B*⋯Cl2	1.091	1.090	2.50	3.546	165
C6—H6*A*⋯O2	1.084	1.089	2.50	3.422	153
C9—H9*A*⋯Cl1	1.089	1.091	2.59	3.472	150
C10—H10*B*⋯Cl2	1.088	1.091	2.75	3.723	157

**Table 5:** Differences (p.p.m.) between selected ^13^C chemical shifts in the solid state and in solution for (1) and (2): (Δ = δ_solution_ − δ_solid_)

	(1)	(2)
No.	Δ	Δ
C2 (C2′)	0.5	−0.8
C3 (C3′)	0.5	−4.1
C6 (C6′)	1.5	2.5
C7	−2.0	−1.0
C7′	−2.0	−1.0
C8	−0.1	0.2
C8′	−0.1	0.2
C9	1.0	1.4
C9′	1.0	1.4
C10	−2.5	−0.6
C10′	−2.5	−0.6

## Footnotes

1 Supporting information for this paper is available from the IUCr electronic archives (Reference: BM5065)
