# Supplementary material for: Structural aspects of intermolecular interactions in the solid state of 1,4-dibenzylpiperazines bearing nitrile or amidine groups
Source: Acta Crystallogr B Struct Sci Cryst Eng Mater. 2014 Sep 18;70(Pt 5):820–7. doi: 10.1107/S2052520614013754 (PMC4184373; doi:10.1107/S2052520614013754)
Supplement: Supplementary file 4 [file b-70-00820-sup4.pdf]

Supporting information

Structural aspects of intermolecular interactions in solid state of 1,4-dibenzylpiperazines bearing nitrile or amidine groups by X-ray diffraction supported by molecular modeling and compared with ^{13}C CP/MAS NMR spectra

Mateusz Rezler, Teresa Żołek, Irena Wolska and Dorota Maciejewska

Table S1. Shielding constants for **1** and **2** (σ, ppm) were calculated at the DFT using the B3LYP/6–311(d, p) basis set.

No.	**1**	**2**
	$\sigma_{calc.}$	$\sigma_{calc.}$
**C1**	36.97	49.53
**C1´**	36.95	49.53
**C2**	53.36	48.24
**C2´**	53.53	48.24
**C3**	48.88	45.87
**C3´**	48.93	45.87
**C4**	68.98	45.66
**C4´**	68.97	45.66
**C5**	49.55	47.29
**C5´**	49.41	47.29
**C6**	54.02	44.98
**C6´**	53.77	44.98
**C7**	64.95	17.04
**C7´**	64.95	17.04
**C8**	116.71	116.25
**C8´**	116.65	116.25
**C9**	129.04	131.27

**C9´**	129.06	131.27
**C10**	125.27	128.31
**C10´**	125.30	128.31

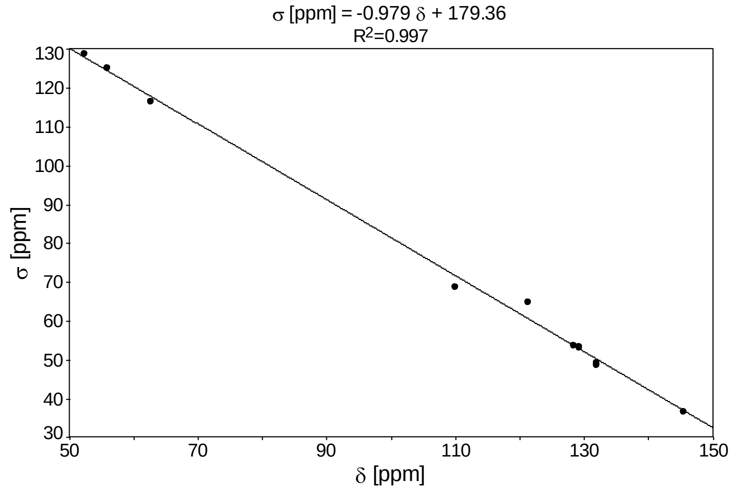

Figure S1. Linear correlation between calculated shielding constants σ and experimental chemical shifts δ for **1**.

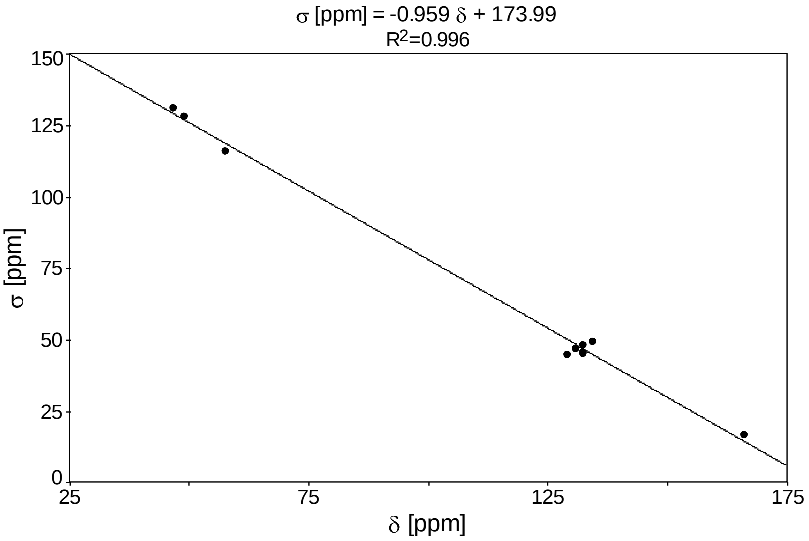

Figure S2. Linear correlation between calculated shielding constants σ and experimental chemical shifts δ for **2**.
